# Sun protection awareness and ultraviolet protection factor product use among pilgrims and nurses during Hajj: A qualitative post-Hajj interview study

**DOI:** 10.1371/journal.pone.0362675

**Published:** 2026-11-11

**Authors:** Omar Qaladi, Fahda Alrasheedi, Manahil Alhawsawi, Raghad Alluhebi, Abeer Abujalid

**Affiliations:** 1 Community and Psychiatric Mental Health Nursing Department, College of Nursing, King Saud University, Riyadh, Saudi Arabia; 2 Madinah Health Cluster, Ministry of Health, Madinah, Saudi Arabia; 3 King Fahd Military Medical Complex Dhahran, Ministry of Defense, Dhahran, Saudi Arabia; 4 Riyadh Second Health Cluster, Ministry of Health, Riyadh, Saudi Arabia; Management and Science University, MALAYSIA

## Abstract

**Background:**

Pilgrims performing Hajj are exposed to extreme heat and ultraviolet (UV) radiation, which pose distinct risks: heat contributes to dehydration and heat-related illness, whereas UV radiation causes sunburn and eye damage and increases long-term skin cancer risk. While heat illness and infectious diseases have been widely studied, awareness and use of UV-protective measures, including sunscreen and ultraviolet protection factor (UPF)-rated clothing and umbrellas, remain underexplored in this context. Understanding both pilgrim and nursing perspectives is essential for developing effective interventions.

**Methods:**

In this qualitative descriptive study, retrospective semi-structured interviews were conducted via Zoom between 20 June and 11 July 2025, within approximately one month after the 2025 Hajj season, with 16 Saudi pilgrims and 13 registered nurses who had worked in hospitals in Makkah and Madinah during Hajj. Interviews explored awareness, attitudes, and behaviors related to UV and sun protection. Data were analyzed thematically following Braun and Clarke’s six-phase approach. Lincoln and Guba’s criteria guided trustworthiness.

**Results:**

Five major themes were identified: (1) physical and emotional impact of sun exposure, (2) limited awareness and knowledge gaps, (3) attitudes and cultural influences on UV/UPF use, (4) barriers and challenges to sun protection, and (5) recommendations and suggestions for improvement. Notably, many participants perceived sun exposure as a spiritual challenge rather than a health risk, UPF-rated products were poorly understood, and practical barriers such as cost, availability, and cultural clothing norms limited use of protection.

**Conclusions:**

The findings highlight critical gaps in awareness and practice regarding sun protection during Hajj. Targeted education before and during Hajj, wider availability of affordable protective items, and culturally appropriate communication strategies are needed, with messages that distinguish heat-related illness from UV-related harm.

## Introduction

The annual Islamic pilgrimage known as Hajj is one of the largest recurring mass gatherings in the world, attracting over two million participants from diverse countries and backgrounds to the holy cities of Makkah and Medina in Saudi Arabia each year [1,2]. Pilgrims are exposed to two related but distinct environmental hazards: intense heat, which increases the risk of dehydration and heat-related illness, and high levels of ultraviolet (UV) radiation, which cause sunburn and eye damage and increase the long-term risk of skin cancer [3,4]. Although the Saudi authorities implement extensive mitigation strategies, including shaded pathways, free distribution of umbrellas and water, and health awareness campaigns, sun protection remains a persistent health challenge during Hajj [2].

Saudi Arabia’s climate, especially during the summer months when Hajj often occurs, exposes pilgrims to high temperatures frequently exceeding 40°C and intense UV radiation, which can lead to conditions such as heat exhaustion, heatstroke, and UV-induced skin damage [5,6]. These hazards call for different protective measures. Sunscreen, rated by its sun protection factor (SPF), and clothing or umbrellas rated by their UPF reduce UV exposure [7,8] whereas hydration, rest, and cooling are central to preventing heat-related illness [9]. Shade from umbrellas offers both thermal and UV benefits, although the UV protection an umbrella provides depends on its fabric [8]. However, awareness and use of these measures among the general public are inconsistent worldwide. Studies show that while awareness of the UV Index or UPF labeling exists, comprehension and translation into protective behavior are often limited [10,11]. A recent study highlighted that 26.9% of pilgrims did not use umbrellas in direct sunlight, suggesting a notable gap in their awareness of associated risks [2].

The global public health community has increasingly emphasized the importance of preventive strategies during mass gatherings, not only for infectious disease control but also for mitigating environmental health risks [12–14]. To address sun-related risks, the World Health Organization and its partners developed the Global Solar Ultraviolet Index (UVI) to promote awareness and guide protective behaviors. However, despite decades of promotion, awareness and practical use of the UVI remain low in many regions [13,14].

In the context of Hajj, health behaviors and preventive practices are shaped by diverse socio-demographic and psychosocial factors, including nationality, education, religious motivation, and access to pre-departure health education [1,15]. Evidence indicates that pilgrims who receive targeted health education prior to travel are more likely to adopt protective practices such as adequate hydration, shade-seeking, and appropriate clothing [9,16,17].

Despite extensive research on infectious diseases and heat illness during Hajj, there is limited evidence on pilgrims’ knowledge, attitudes, and behaviors regarding UV protection and UPF-rated products. Moreover, cultural beliefs, language barriers, and logistical constraints may influence how pilgrims and healthcare providers approach sun safety. Therefore, this study seeks to explore pilgrims’ and nurses’ perspectives on sun protection and the use of sun-protective products, including sunscreen and UPF-rated clothing and umbrellas, during Hajj. By identifying awareness levels, cultural influences, and practical barriers, the study aims to inform tailored educational strategies and health interventions that reduce heat- and UV-related risks in future Hajj seasons, and to support safer working conditions for nurses deployed at mass gatherings.

## Materials and methods

### Study design and aims

This study employed a qualitative descriptive design using semi-structured interviews to explore awareness, attitudes, and behaviors related to sun protection and the use of sun-protective products among Hajj pilgrims and nurses. A qualitative descriptive design with inductive thematic analysis was chosen over a framework-driven approach (e.g., Health Belief Model, Protection Motivation Theory) given the largely unexplored nature of sun protection behaviors in the Hajj context. An inductive approach allowed themes specific to this setting, including spiritual reframing of physical hardship and Ihram-related clothing constraints, to emerge without being constrained to predetermined behavioral constructs. Because interviews took place after Hajj, the study captures participants’ recalled experiences and self-reported practices rather than directly observed behavior. The study is reported in accordance with the Consolidated Criteria for Reporting Qualitative Research (COREQ) (S1 Checklist).

### Setting and sample

The study focused on experiences in Makkah and Madinah, the two central locations of the Hajj pilgrimage in the Kingdom of Saudi Arabia, where participants were recruited. Interviews were conducted remotely via Zoom from 20 June to 11 July 2025, within approximately one month after the conclusion of the 2025 Hajj season, to limit the time between the experience and participants’ recollection of it. Participants were therefore asked to recall their experiences of intense heat, dense crowds, and physically demanding rituals; they were not observed or interviewed during the rituals themselves.

A purposive sampling strategy was adopted to recruit targeted participants. The first sample group included Saudi pilgrims who had completed the Hajj pilgrimage within one month prior to the interview. Focusing on Saudi pilgrims was an intentional sampling decision for this exploratory study, allowing greater linguistic and cultural consistency within the pilgrim group and providing a context-specific baseline before extending future research to more culturally and nationally diverse international pilgrim populations. Following permission from the relevant recruitment sites, a member of the research team directly approached potential pilgrim participants at accommodation centers, community mosques, and Hajj support facilities in Makkah and Madinah within approximately one week after Hajj. Pilgrims were approached based on their ability to describe their personal experiences, behaviors, and decisions regarding sun exposure and protective strategies during the pilgrimage. Individuals who expressed interest provided their contact details so that a subsequent Zoom interview could be arranged. The second sample group consisted of registered nurses who had provided direct clinical care to pilgrims during the 2025 Hajj season. Following official permission from the Ministry of Health and participating hospitals, a member of the research team directly approached eligible nurses. Hospital and Ministry of Health managers authorized the researchers to approach potential participants but did not select or invite nurses and were not informed of who agreed or declined to participate. Nurses who expressed interest provided contact details so that a subsequent Zoom interview could be arranged. For both groups, participation was voluntary, and informed consent was obtained before the interview. These nurses offered professional insights into sun protection practices observed among pilgrims, challenges encountered when disseminating health education in mass gathering contexts, and institutional preparedness for managing heat-related health concerns. Inclusion criteria for both groups required participants to be 18 years of age or older and directly involved in the 2025 Hajj season. Individuals who had not been involved in the 2025 Hajj season or were unable to provide informed consent were excluded.

In qualitative research, the determination of sample size is shaped by the study’s purpose, philosophical assumptions, and the need for in-depth exploration of the phenomenon of interest [18,19]. Inadequate sample sizes may compromise the richness and credibility of the findings [19]. Achieving thematic saturation, the point at which no new concepts emerge and data begin to repeat, is essential to ensuring analytical completeness [18]. Because pilgrims and nurses were expected to offer different perspectives, saturation was assessed separately for each group. Interviews were reviewed and coded as they were completed, and the team checked whether each new interview added new codes. Saturation was judged to have been reached in each group when the final two to three interviews in that group produced no new codes; this occurred after 16 interviews with pilgrims and 13 with registered nurses. The resulting sample provided sufficient depth to address the study aims as a purposive qualitative sample.

### Data collection

Data were collected through semi-structured, in-depth interviews using an interview guide developed through a review of relevant literature and consultation with experts in nursing, public health, and qualitative research. The guide was designed to explore participants’ awareness, behaviors, and attitudes related to sun protection during the Hajj pilgrimage, including their familiarity with UPF-labeled products, responses to extreme heat exposure, and perceived challenges and facilitators to sun-safe practices. The finalized set of interview questions was applied to both participant groups, as shown in Table 1.

**Table 1:** The interview questions used with both groups of participants.

1	For Both Pilgrims and Nurses participants
1.2	Describe your experience with sun exposure during Hajj, how did it affect your comfort or health?
1.3	Have you used any sun protection products such as umbrellas, sunscreens, or long-sleeved clothing during Hajj? And when purchasing these products, did you check whether they offer ultraviolet protection (UPF)?
1.4	What is your understanding of how these products protecting you from health problems caused by sun exposure?
1.5	What are your opinions or beliefs about using sun protection such as sunscreen or umbrellas during Hajj?
1.6	What challenges did you face in using sun protection? (e.g., cost, difficulty in accessing, lack of awareness, cultural customs, etc.)
2	For Pilgrim Participants Only
2.1	Where did you get your information about sun protection during Hajj? (e.g., healthcare staff, media, friends, religious guides, etc.)?
2.2	Did any healthcare workers provide you with advice on sun protection? If yes, what kind of advice?
2.3	Would you like more information or guidance about sun protection before or during Hajj? How would you prefer to receive it?
3	For Nurses Participants Only
3.1	Have you ever received any formal training or education related to sun protection strategies for pilgrims? If yes, describe this training.
3.2	In your experience, to what extent do pilgrims typically adhere to sun protection instructions such as the use of UPF sunscreens or umbrellas?
3.3	What are the most significant challenges or obstacles you face in raising awareness or promoting the use of sunscreen among pilgrims?
4	For Both Pilgrims and Nurses participants
4.1	Do you have any suggestions or recommendations for improving awareness and use of sunscreen during the Hajj season

The interview guide was reviewed by a panel of experts to ensure clarity, cultural appropriateness, and relevance to the study aims. Pilot testing was conducted with one pilgrim and one nurse, and minor revisions were made to enhance question flow and clarity.

All interviews were conducted via Zoom by a trained female researcher (MA). MA received formal training in qualitative research methodologies, including interview-based data collection, to ensure methodological rigor and consistency throughout the process. She had no prior relationship with the participants, which helped to promote impartiality and reduce the risk of interviewer bias. Participants joined the interviews from their homes, no one other than the participant and the interviewer was present, and each participant was interviewed once. During the consent process, participants were informed of the interviewer's professional background and the aims of the study.

All pilgrims and all Saudi nurses were interviewed in Arabic. The single non-Saudi participant, a nurse, was interviewed in English, her primary professional and working language. To ensure the preservation of meaning during translation, the research team followed the guidelines proposed by Van Nes et al. for translating qualitative data [20].

All interviews were audio-recorded with the participants’ consent and lasted approximately 30–45 minutes. Field notes were taken during and after each interview to document contextual details and early analytical impressions. Recordings were transcribed verbatim in the original interview language. Arabic transcripts were translated into English by a bilingual scholar and MA, both fluent in Arabic and English. To verify translation quality, a second bilingual researcher, independent of the original translation, audited a random 20% sample of translated transcripts and quotations against the original Arabic recordings to confirm conceptual and semantic equivalence. All participants were invited to review their transcripts to confirm accuracy as part of the member-checking process. No participants withdrew from the study during data collection.

#### Researcher reflexivity.

Interviews were conducted by MA, a senior registered nurse with 8 years of clinical experience in Emergency Department and Medical-Surgical settings. This clinical background facilitated rapport with participants and contextual understanding of their accounts but also carried a risk of interpreting participant narratives through a clinical lens rather than participants’ own framing. To mitigate this, MA engaged in bracketing prior to data collection to document her personal assumptions about sun protection and Hajj health behaviors. Interviews strictly followed the semi-structured, open-ended guide to limit interviewer-driven probing, and MA maintained a reflexive journal after each session, noting interpersonal dynamics and her own reactions. During analysis, investigator triangulation (independent coding by multiple researchers followed by peer debriefing) helped ensure that themes remained grounded in participants’ accounts rather than the interviewer's clinical perspective.

### Data analysis

Interview transcripts were imported and analyzed using NVivo 12.6 to facilitate systematic coding and theme development. Three researchers independently coded an initial 20% sample of transcripts to develop a shared codebook; the remaining transcripts were then coded independently by each researcher against this codebook. Coding discrepancies were resolved through consensus-building discussion among the coding team, with a senior researcher acting as arbiter where agreement could not be reached. Thematic analysis followed Braun and Clarke's six-phase approach, which includes: familiarization with the data, generating initial codes, searching for themes, reviewing themes, defining and naming themes, and producing the final report [21]. The analysis process was iterative and reflexive, allowing for themes to evolve as coding progressed. Initial codes were grouped into broader categories based on conceptual similarity, and relationships between categories were mapped to identify candidate themes. Candidate themes were then merged, refined, or discarded through team discussion until five distinct, non-overlapping themes emerged that comprehensively represented the dataset.

To ensure the trustworthiness of the findings, the study adhered to Lincoln and Guba’s criteria for qualitative rigor, including credibility, dependability, confirmability, transferability, and authenticity [22]. Credibility was enhanced through member checking, where participants were invited to review and verify their transcribed interviews. Dependability and confirmability were supported by detailed audit trails and regular peer debriefings among the research team. To improve transferability, comprehensive descriptions of the research setting, participant demographics, and context were provided. Authenticity was prioritized by preserving the richness and nuance of participant experiences, using direct quotations where appropriate to illustrate each theme.

Themes were refined collaboratively through regular meetings among the research team to reach consensus and ensure that interpretations were grounded in the data. The final themes represent both shared and divergent perspectives among participants and reflect the complex interplay of knowledge, beliefs, and behaviors related to sun protection and UV exposure during Hajj (Table 2).

**Table 2:** Summary of major themes and subthemes of the qualitative study results.

Major Themes	Subthemes
1. Physical and Emotional Impact of Sun Exposure	1.1 Heat Stress Symptoms 1.2 Impact on Mood and Mental State 1.3 Vulnerable Groups
2. Limited Awareness and Knowledge Gaps	2.1 Basic knowledge of sun risks 2.2 Lack of understanding of UV/UPF concepts 2.3 Misunderstanding the Effectiveness of Protective Tools
3. Attitudes and Cultural Influences on UV/UPF Use and Sun Protection Practices	3.1 Perceived Necessity and Daily Practices 3.2 Cultural Beliefs and Social Norms
4. Barriers and Challenges in Using Sun Protection	4.1 Communication issues 4.2 Practical Limitations and Physical Discomfort 4.3 Access, Cost, and Cultural Clothing Constraints
5. Recommendations and Suggestions for Improvement	5.1 Education Before and During Hajj 5.2 Improving Access to Protective Items 5.3 Product Design and Cultural Fit

### Ethics approval and consent

This study complied with the Declaration of Helsinki and received ethics approval from the Institutional Review Board of King Salman bin Abdulaziz Medical City (IRB No. H-03-M-11), which is responsible for overseeing research conducted under Ministry of Health (MOH) facilities in Makkah and Madinah. Informed consent was obtained from all study participants, who were informed of their right to withdraw at any time or skip any questions during the interview. Participant anonymity and confidentiality were strictly maintained throughout the study.

## Results

### Participants’ demographic information

A total of 29 participants were interviewed, comprising 16 pilgrims and 13 registered nurses. All pilgrims were Saudi nationals (seven men and nine women; mean age 37 years). Nurses comprised five men and eight women (mean age 33 years), and 12 of the 13 nurses were Saudi nationals (Table 3).

**Table 3:** Characteristics of interview participants.

Category	Interview participants (n = 29)	
	Pilgrims n = 16 (%)	Nurses n = 13 (%)
**Gender**		
Male	7 (44%)	5 (38%)
Female	9 (56%)	8 (62%)
**Age (Mean)**	37	33
**Marital status**		
Single	5 (31%)	3 (23%)
Married	11 (69%)	10 (77%)
**Nationality**		
Saudi	16 (100%)	12 (92%)
Non-Saudi	0 (0%)	1 (8%)

### Physical and emotional impact of sun exposure

Participants described the physical and emotional burden of sun exposure during Hajj. Their accounts reflect not only the immediate physiological effects of the intense heat but also its influence on psychological well-being and spiritual endurance. Notably, participants described this burden almost entirely in terms of heat: the symptoms reported (exhaustion, dizziness, dehydration, and fainting) are heat-related, whereas UV-specific effects such as sunburn or eye irritation were rarely raised. This theme comprises three subthemes: heat stress symptoms, impact on mood and mental state, and experiences among vulnerable groups.

#### Heat stress symptoms.

Both pilgrims and nurses described heat-related symptoms during prolonged outdoor exposure, including feeling physically drained, dizziness, dehydration, and near-fainting during outdoor rituals. One pilgrim shared, *“The weather was hot, and, unfortunately, the sun was directly overhead... We felt tired and exhausted you feel like you can’t continue the remaining steps.”* Another pilgrim listed: *“Dehydration, dizziness and fainting.”* A nurse confirmed these experiences were not isolated, stating, *“Some of my colleagues and pilgrims got heat exhaustion and fainted.”* These accounts describe heat-related illness rather than UV-related harm and point to the need for heat-prevention measures during high-risk periods.

#### Impact on mood and mental state.

The psychological toll of sun exposure emerged as another significant concern. Participants noted that excessive heat influenced their mood, often leading to irritability, fatigue, and stress. One nurse reflected, *“The heat does affect the mood somewhat... you come back feeling tired, exhausted, sometimes it even reaches the point of stress.”* Another nurse described how the heat affected interpersonal dynamics: " *People get more tense and short-tempered when it’s hot. I noticed even among us nurses, we were less patient with each other”*. Nonetheless, several pilgrims emphasized spiritual resilience and drew upon faith to maintain a positive outlook. A recurring phrase among pilgrims was *“Alhamdulillah” (praise be to God)*, reflecting their effort to reframe discomfort within a religious context. This attitude appeared to buffer emotional distress for some individuals.

#### Vulnerable groups.

Elderly pilgrims and those with chronic conditions such as hypertension and diabetes were repeatedly described as particularly affected by the heat. Participants described physical struggles and the need for additional care. One noted, *“The elderly suffer a lot... They can't handle the sun. They get tired quickly, and some need wheelchairs to move between places.”* Another recalled, *“I remember one old woman with hypertension, she couldn’t continue the rituals in the heat, she had to stop and rest every few minutes.”*

Nurses emphasized the importance of monitoring these individuals, with one stating, *“We try to stay close to the older pilgrims. They don’t always speak up, but you can tell they are struggling with the sun.”* These reflections underscore the heightened risks faced by vulnerable groups and the protective role played by nurses.

### Limited awareness and knowledge gaps

Participants generally showed basic awareness of sun risks and protective measures but limited understanding of specialized concepts such as UPF and the UV index. They readily associated the sun with heat-related illness, but knowledge of how UV radiation specifically affects the skin and eyes was limited. Three subthemes emerged under this theme.

#### Basic knowledge of sun risks.

Participants commonly mentioned staying hydrated and avoiding direct sun, but their knowledge was based on informal tips rather than structured education. One pilgrim reported *“I didn’t receive formal training... just messages like “avoid the sun and drink water.”* Another pilgrim said, *“Everyone knows the sun is harmful, but I don’t think we understand how exactly.”* A registered nurse added, *“We were told the basics, but nothing detailed such as about UV protection and how it actually affects the body.”* This highlights a tendency to follow general advice in the absence of deeper awareness.

#### Lack of understanding of UV/UPF concepts.

Limited familiarity with specialized terms such as UPF and the UV index was evident in both groups. For instance, one of the nurses stated *“Honestly, I have no knowledge about UV-protection products”*, while another nurse remarked that it was the *“first time I heard about UPF”*. Pilgrims, likewise, often described hearing of UV-protective products for the first time during the interview. One of the pilgrims stated, *“I didn’t even know there are special fabrics for blocking UV rays.”* These responses highlight the novelty of such concepts for participants in both groups, despite their relevance to sun safety during Hajj.

#### Misunderstanding the effectiveness of protective tools.

Participants in both groups were often unaware of the difference between ordinary and UPF-rated products, assuming all physical barriers offered equal protection. One nurse stated *“I always thought any umbrella would do the job. I never realized that there are umbrellas with special materials to block UV rays.”*. A pilgrim said, *“We wear abayas and cover our heads, but I didn’t know the fabric could make a difference.”* These misconceptions highlight the gap between intention and actual effectiveness in sun protection practices.

### Attitudes and cultural influences on UV/UPF use and sun protection practices

This theme examines how personal beliefs, cultural norms, and religious values shape attitudes and behaviors toward sun protection and the use of UPF-rated products during Hajj.

#### Perceived necessity and daily practices.

Participants in both groups generally viewed sun protection as essential for safely completing Hajj rituals under extreme heat. A nurse stated “We see the effects of the sun every year without protection, people get sick quickly. Using umbrellas or sunscreen is not optional; it’s necessary.” Pilgrims echoed this view: “We all carried umbrellas. Even if it’s uncomfortable, it’s better than getting sick.” After learning about UPF-rated products, several pilgrims expressed interest: “If someone had explained about UPF umbrellas or clothing before Hajj, I would have definitely brought them along”

#### Cultural beliefs and social norms.

Cultural and religious values played a role in shaping attitudes toward sun protection. Participants described how some pilgrims, especially older pilgrims, viewed sun exposure as part of the spiritual hardship of Hajj. A nurse explained, *“Some elderly pilgrims believe enduring the sun is part of the spiritual challenge; they think the harder it is, the greater the reward.”* Similarly, a pilgrim shared, *“Some people feel they should face the heat as part of the sacrifice of Hajj. They even refuse to use umbrellas or hats.”*

Sunscreen use was described mainly by female pilgrims. One noted, “*I use sunscreen because it has no perfume because perfume is forbidden during Ihram.*”. Male nurses and pilgrims, by contrast, generally reported little or no sunscreen use: “*No sunscreen, no hats … just got an umbrella if the weather got extremely hot*,”.

At the same time, participants described a noticeable shift in public perception. Sun protection practices that were once uncommon or stigmatized are now widely accepted. As one pilgrim noted, *“In the past, you wouldn’t see many people carrying umbrellas, maybe they felt shy. But now its common umbrellas are everywhere.”* A nurse added, *“There’s more acceptance now. People understand the importance and don’t feel embarrassed to use protection like before.”*. These findings highlight how culture and spiritual interpretations can influence personal decisions, even at the expense of physical health.

### Barriers and challenges in using sun protection

Despite recognizing the importance of sun protection, participants reported several obstacles that hindered its consistent use. These barriers were both practical and systemic, highlighting a gap between intention and practice.

#### Communication issues.

Nurses described reported language differences as a major barrier when educating diverse pilgrim groups. Many pilgrims did not speak Arabic or English, and communication was further complicated when individuals had hearing or speech impairments. One nurse noted, “*The biggest challenge is language specifically who can't understand Arabic or English … also some pilgrims are deaf, so we must have a sign-language translator*.” Language barriers also operated in the other direction; a nurse who did not speak Arabic described difficulty communicating with Saudi pilgrims: “*It’s difficult when the pilgrim is Saudi and can’t understand English. I can't explain in Arabic, I feel stuck sometimes”*. These communication issues created challenges in providing effective sun protection instructions during time-sensitive and crowded situations.

#### Practical limitations and physical discomfort.

Even when pilgrims intended to use sun protection, various practical issues interfered with consistent use. Participants reported forgetting or misplacing items like umbrellas during the busy Hajj schedule. One pilgrim shared: *“I forgot my umbrella at the camp… and I couldn’t find anyone selling or handing out umbrellas on the way.”* A nurse explained: *“Some older pilgrims forget their umbrellas in the hotel room, then go out without any shade.”*

In addition to forgetfulness, some pilgrims found certain protective items inconvenient to use. For instance, holding heavy or bulky umbrellas for long periods in crowded conditions proved physically exhausting: *“The umbrella was a bit cumbersome… the one I bought was heavy; carrying it for an hour or two made me tired.”.* These factors illustrate how physical burden and situational constraints can reduce consistent sun protection, even among those with good intentions.

#### Access, cost, and cultural clothing constraints.

While basic sun protection tools such as regular umbrellas were generally available and sometimes distributed free of charge, access to more specialized products such as UPF-rated umbrellas, clothing, or caps was limited. As one pilgrim explained, “*I thought it would be like 200 riyals… but then someone told me you can get it online for 40 riyals only*.” Another pilgrim reported that they “*didn’t know where to find this kind of umbrella with UV protection before Hajj*”.

Cultural dress codes for women added to the challenge. Female nurses and pilgrims described additional heat stress from clothing that was not designed for hot, sunny conditions. A nurse stated, “*Even if I have an umbrella, the abaya makes it worse... I feel the sun hitting harder*”. Another nurse described the lack of uniforms or abayas suitable for working long hours under direct sun: “*Our abayas and uniforms are not made of breathable or UV-protective material, …. also, there’s no awareness or access to abayas made for this purpose, you won’t find them easily in the market.*”

### Recommendations and suggestions for improvement

Participants offered practical and context-specific suggestions to enhance sun protection practices during Hajj. Their recommendations reflected lived experiences and addressed systemic, educational, and logistical gaps.

#### Education before and during Hajj.

Participants in both groups stressed the need for structured education targeting both pilgrims and nurses. Pre-Hajj awareness campaigns were seen as especially important. One pilgrim stated: “*If I had known about UV umbrellas or cooling clothes before, I would’ve brought them for sure,”.* Nurses also suggested delivering health tips in pilgrims’ native languages before arrival: *“Education should start early, in the home country, with clear messages about heat, dehydration, and sun protection, UV protection”.*

#### Improving access to protective items.

Participants recommended making sun protection tools, especially UPF-rated items, more visible and accessible on-site. One nurse stated, “*We should have more shops or vending machine distributing proper umbrellas or cooling gear in key locations”.* Another nurse proposed including UPF-rated items in official Hajj kits: *“If they gave every pilgrim a proper UV umbrella and explained its benefit, more people would actually use it”.*

#### Product design and cultural fit.

Several female participants emphasized the need for breathable, culturally appropriate clothing designed for heat protection. One nurse stated “*We wear thick abayas or long uniforms, which make the heat worse. There should be lighter materials that still meet modesty rules*,”.

### Themes summary

Overall, pilgrims and nurses largely converged in recognizing sun exposure as a serious but under-addressed risk during Hajj. Differences were most apparent in the *Limited Awareness and Knowledge Gaps* theme: nurses more often expressed general awareness of UV protection, although they had received no formal training on it, and saw themselves as responsible for educating pilgrims. In the *Barriers* theme, nurses emphasized systemic and communication obstacles (e.g., language barriers with international pilgrims and lack of formal training), whereas pilgrims emphasized personal and practical obstacles (e.g., cost, comfort, and forgetting items). Both groups converged strongly in the *Recommendations* theme, independently proposing pre-travel education as the highest-priority intervention.

## Discussion

This study explored the awareness, perceptions, and sun protection practices of pilgrims and registered nurses during the Hajj pilgrimage, with a particular focus on the use of sunscreen and UPF-rated products and on UV-related health behaviors. The findings reveal important gaps in sun safety awareness and practice among both groups, despite growing environmental risks and available public health resources. Given that sun protection and UPF awareness during Hajj remain almost entirely unstudied qualitatively, we employed an inductive, data-driven thematic approach rather than structuring the analysis around an a priori behavioral framework such as the Health Belief Model or Protection Motivation Theory, allowing culturally and contextually specific themes to emerge on their own terms. This leaves the findings largely descriptive rather than explanatory of underlying behavioral mechanisms; future confirmatory research could usefully apply an established behavioral framework to test the relationships suggested by these themes.

Both nurse and pilgrim, described a significant physical discomfort associated with prolonged sun exposure during Hajj, including fatigue, dizziness, and symptoms of dehydration. These experiences are consistent with prior studies highlighting the high incidence of heat-related illness among pilgrims [1,2]. In addition to physical symptoms, several participants reported emotional strain, such as irritability, fatigue, and stress. Environmental stressors not only disrupted ritual performance but also contributed to psychological distress, particularly among older adults and those with chronic illnesses. These findings align with previous research emphasizing the burden of physical and emotional stress during pilgrimage in high-heat conditions [2,10]. Addressing these effects requires integrative strategies that consider both physiological protection and emotional well-being.

An important pattern across these accounts is that participants understood sun exposure largely as a heat problem. The symptoms they described (exhaustion, dizziness, dehydration, and fainting) are manifestations of heat stress, which is driven by air temperature, humidity, radiant heat, and exertion, whereas UV radiation causes sunburn, eye injury, and cumulative skin damage that is not necessarily felt as heat [4,11,23]. The two hazards share a source but differ in prevention: hydration, rest, and cooling address heat-related illness; sunscreen and UPF-rated clothing and umbrellas address UV exposure; and shade contributes to both. Conflating the two may lead pilgrims to judge their protection by thermal comfort, for example assuming that any umbrella or covering garment is adequate, as several participants did, even though the UV protection of fabrics and umbrella canopies varies [7,8]. Health messages during Hajj may therefore need to name heat and UV as separate risks, each with its own protective actions.

Findings from this study indicate that awareness of sun-related health risks and protective strategies remains limited among participants in this study. Despite exposure to intense heat, few pilgrims demonstrated familiarity with UV-related concepts such as UPF-rated clothing or the UVI, a pattern reported in global studies highlighting insufficient public engagement with sun safety tools [3,14,23]. Participants’ knowledge came largely from informal messages rather than structured pre-travel education, which is consistent with previous research linking health education to better preventive behaviors [1]. Moreover, registered nurses indicated that sun protection specifically related to UV protection received minimal emphasis in both clinical training and health promotion activities, which tended to prioritize communicable disease prevention. This imbalance, also noted in previous studies, may leave nurses less prepared to counsel pilgrims on UV protection [13,14]. These findings highlight the importance of incorporating environmental health content into pre-Hajj education for both nurses and pilgrims to foster more comprehensive and preventive sun safety practices during mass gatherings.

Cultural and religious beliefs emerged as powerful determinants of sun protection behaviors during Hajj, revealing complex interactions between spiritual interpretations and health practices. Participants described how some elderly pilgrims viewed sun exposure as an integral component of the spiritual challenge inherent to Hajj, believing that enduring physical hardship would lead to greater spiritual reward. This perception reflects broader patterns in religious communities where physical suffering is interpreted as spiritually meaningful, potentially leading to reduced motivation for protective health measures [24,25]. Such attitudes demonstrate how religious coping strategies can sometimes conflict with health-promoting behaviors, particularly when hardship is viewed as a pathway to spiritual growth [24,25]. However, the study also revealed positive cultural adaptations, with participants describing a notable shift in social acceptance of sun protection practices. Participants observed that umbrella use, once considered uncommon or stigmatized, had become widely accepted and normalized among pilgrims, suggesting an evolving cultural norm that increasingly embraces protective behaviors. This evolution indicates that cultural beliefs are not static barriers but can adapt to incorporate health-promoting practices when appropriately contextualized within existing value systems [24,25]. These findings underscore the importance of developing culturally responsive health interventions that work within, rather than against, existing belief systems to promote sun safety during mass religious gatherings.

This study identified multiple interconnected barriers to sun protection during Hajj, reflecting the complex challenges of promoting health behaviors in mass gathering contexts. Communication barriers emerged as particularly significant, with nurses reporting difficulties in providing effective health education to linguistically diverse pilgrims, a finding consistent with previous research showing language as a major obstacle to healthcare delivery in multicultural settings [26–28]. Practical constraints were also highlighted, including forgetting protective items amid demanding schedules and the physical burden of carrying umbrellas in crowded areas, patterns similarly noted in recent Hajj health studies [2,9,16]. Misconceptions regarding the cost and availability of UPF-rated products further influenced practices, with some participants overestimating expenses and underestimating accessibility, suggesting that perceived barriers may be as influential as actual ones. Cultural clothing requirements created additional constraints, particularly for women, as traditional dress both intensified heat stress and limited adaptation options, in line with findings from Saudi research on sun protection [10]. Overall, these findings underscore the need for comprehensive interventions that simultaneously address communication, practical, economic, and cultural barriers.

Participants’ recommendations for enhancing sun protection during Hajj revealed a comprehensive understanding of the multi-level interventions required to address identified barriers and knowledge gaps. The most prominent suggestion was implementing structured pre-travel education programs that would target both pilgrims and healthcare providers in their countries of origin, reflecting recognition that effective prevention requires preparation rather than reactive measures during the pilgrimage itself. This finding aligns with established evidence demonstrating that pre-travel health education significantly improves preventive behavior adoption among international travelers and pilgrims [1,17]. Participants also highlighted the critical need for multilingual educational materials and culturally appropriate communication strategies, addressing the significant language barriers that nurses reported as major obstacles to effective health education during Hajj.

## Conclusion

This study highlighted key gaps in sun protection awareness and practices among pilgrims and registered nurses experienced during Hajj. Despite recognition of environmental risks, the use of preventive measures was limited due to cultural beliefs, logistical barriers, and inadequate education. The findings underscore the need for integrated, culturally appropriate strategies, including structured pre-travel education, improved access to sunscreen and UPF-rated clothing and umbrellas, and multilingual resources to enhance sun safety during Hajj. Since participants largely equated sun exposure with heat, health messages should distinguish heat-related illness from UV-related harm, as each requires different protective actions.

## Strengths and limitations

This study provides one of the first qualitative examinations in the Hajj context of UV and UPF awareness, attitudes, and practices among pilgrims and registered nurses. Interviewing participants within approximately one month after Hajj, while their experiences were still recent, allowed rich, context-specific insights into the cultural, environmental, and organizational factors influencing sun protection. Including both pilgrims and the nurses who cared for them provided complementary perspectives on the same events. Its focus on the distinctive cultural, environmental, and organizational context of Hajj enhances the applicability of the findings to public health strategies and sun safety initiatives within similar mass gathering settings.

However, the study was conducted with a single nationality of pilgrims during one Hajj season, which may limit the transferability of findings to broader, more diverse pilgrim populations. The inclusion of Saudi pilgrims only may also have influenced the types of barriers and perceptions identified. In particular, the language barriers reported in this study primarily reflect nurses’ experiences caring for the wider multinational Hajj population rather than communication difficulties experienced by the Saudi pilgrim participants themselves. Cultural beliefs, socioeconomic circumstances, familiarity with sun-protection practices, and access to sun-protection products may also differ among pilgrims from other countries. Accordingly, these findings should not be assumed to represent the experiences of the broader international Hajj population. Qualitative design, while valuable for depth of understanding, captures subjective perceptions that may not represent the full range of experiences or allow for statistical generalization. Despite these constraints, the study establishes a foundation for developing culturally tailored interventions to improve sun protection in mass gathering settings.

Additionally, as with any interview-based study on health behaviors, responses may be subject to social desirability bias, particularly around admissions of non-adherence to protective measures. Recall bias is also possible, as interviews took place up to several weeks after participants’ Hajj experience. Finally, conducting interviews via Zoom rather than in person may have affected rapport-building and the richness of nonverbal cues, though no participants reported technical or communication difficulties during interviews. Education level, prior Hajj experience, nurses’ years of professional experience, and socioeconomic status were not captured in this study's demographic data and may have offered further explanatory context.

## Implications

The findings of this study highlight the need to integrate sun protection into broader health strategies during Hajj. Public health authorities should prioritize culturally sensitive education on UV exposure and heat-related illness, particularly during pre-travel preparations for Saudi pilgrims. Health promotion materials should be multilingual, visual, and accessible, addressing gender norms, spiritual beliefs, and practical barriers to implementation. Given the influential role of registered nurses, their training should include structured content on environmental health risks, with emphasis on sun safety counseling. In addition, ensuring the availability and affordability of sun-protective products such as sunscreen, UPF-rated umbrellas, and protective clothing is essential. Participants’ suggestions, such as installing accessible points of distribution for sun-protective products (e.g., sunscreen, UPF-rated foldable umbrellas, cooling towels, UV-protective sleeves) in high-exposure areas, offer a starting point worth piloting and evaluating rather than a validated solution; these ideas emerged from participant recommendations rather than being tested within this study and should be assessed for feasibility and impact before wider implementation. Addressing sun protection as a public health priority during Hajj can contribute to reducing preventable morbidity and enhancing pilgrims’ overall safety and well-being.

## Supporting information

S1 Checklist COREQ checklist. Completed Consolidated Criteria for Reporting Qualitative Research (COREQ) 32-item checklist, with the location of each item in the manuscript.
(DOCX)

## Abbreviations

UV: ultraviolet
UPF: ultraviolet protection factor
SPF: sun protection factor
UVI: ultraviolet Index.
