# Supplementary material for: Sun protection awareness and ultraviolet protection factor product use among pilgrims and nurses during Hajj: A qualitative post-Hajj interview study
Source: PLoS One. 2026 Nov 11;21(11):e0362675. doi: 10.1371/journal.pone.0362675 (PMC13754106; doi:10.1371/journal.pone.0362675)
Supplement: Supplementary file 1 [file pone.0362675.s004.pdf]

Sampling strategy

The study included only Saudi pilgrims, despite Hajj being an international mass gathering. From my perspective the restriction might have limits transferability. kindly justify why only Saudi pilgrims were included and explain if this limitation may have influenced findings regarding language barriers, cultural beliefs, and access to sun protection.

Please provide additional detail regarding **participant recruitment**, including how potential participants were approached, who made the initial contact, whether participation was voluntary without institutional influence. This information is particularly relevant because nurses were recruited through the Ministry of Health and participating hospitals.

The authors' claim of "representative account" should be removed. For a purposively sampled qualitative study involving **16 pilgrims and 13 nurses**, "representative" is problematic.

COREQ reporting considerations

Given that this is a qualitative interview study, the authors are encouraged to assess the manuscript against the Consolidated Criteria for Reporting Qualitative Research (COREQ) 32-item checklist. Several COREQ domains, particularly researcher reflexivity, participant recruitment, saturation, data collection procedures, and analytic processes, have been addressed in the revision; however, the manuscript should be checked systematically for completeness. If appropriate, please provide the completed COREQ checklist as a supplementary file

In the limitation section

Primary data are retrospective interviews, this should be integrated into the interpretation rather than merely listed as a limitation. The title/abstract should also avoid implying that the researchers directly observed behaviour during Hajj.

Overall

The authors have responded constructively to the previous comments. However, several issues remain that should be addressed before the manuscript can be considered for publication. Most importantly, the manuscript contains an internal inconsistency regarding the timing of data collection, with the Methods indicating retrospective Zoom interviews after Hajj while the Strengths section states that data were collected in real time during the pilgrimage. In addition, the manuscript should distinguish more carefully between heat-related illness and UV-related health effects and correct the terminology surrounding UPF and sunscreen. Further clarification is required regarding saturation across the two participant groups, recruitment procedures, and the evidentiary basis for statements such as “most participants.” Kindly address these issues.
