# Supplementary material for: Sun protection awareness and ultraviolet protection factor product use among pilgrims and nurses during Hajj: A qualitative post-Hajj interview study
Source: PLoS One. 2026 Nov 11;21(11):e0362675. doi: 10.1371/journal.pone.0362675 (PMC13754106; doi:10.1371/journal.pone.0362675)
Supplement: Supplementary file 2 [file pone.0362675.s001.docx]

**S1 Checklist. COREQ (Consolidated criteria for reporting qualitative research) checklist**

*Manuscript: Sun protection awareness and ultraviolet protection factor product use among pilgrims and nurses during Hajj: a qualitative post-Hajj interview study*

| **No.** | **Item** | **How reported** | **Location in manuscript** |
| --- | --- | --- | --- |
| **Domain 1: Research team and reflexivity** | | | |
| ***Personal characteristics*** | | | |
| 1 | Interviewer/facilitator | MA conducted all interviews. | Methods – Data Collection; Researcher Reflexivity |
| 2 | Credentials | Registered nurse; MSN. | Methods – Researcher Reflexivity |
| 3 | Occupation | Senior registered nurse (Emergency Department and Medical-Surgical settings); co-author and member of the study team. | Methods – Data Collection; Researcher Reflexivity |
| 4 | Gender | Female. | Methods – Data Collection |
| 5 | Experience and training | Formal training in qualitative research methods, including interview-based data collection; 8 years of clinical experience. | Methods – Data Collection; Researcher Reflexivity |
| ***Relationship with participants*** | | | |
| 6 | Relationship established | No prior relationship with participants. | Methods – Data Collection |
| 7 | Participant knowledge of the interviewer | Participants were informed of the interviewer’s identity, professional background, and the study aims during the consent process. | Methods – Data Collection |
| 8 | Interviewer characteristics | Clinical background, potential for interpretation through a clinical lens, bracketing, reflexive journal, investigator triangulation. | Methods – Researcher Reflexivity |
| **Domain 2: Study design** | | | |
| ***Theoretical framework*** | | | |
| 9 | Methodological orientation and theory | Qualitative descriptive design; inductive thematic analysis (Braun and Clarke); rationale for not using an a priori behavioral framework. | Methods – Study Design and Aims; Data Analysis |
| ***Participant selection*** | | | |
| 10 | Sampling | Purposive sampling. | Methods – Setting and Sample |
| 11 | Method of approach | Approached in person by a member of the research team (pilgrims at accommodation centers, mosques, and Hajj support facilities; nurses after MOH/hospital permission); interviews arranged via Zoom. | Methods – Setting and Sample |
| 12 | Sample size | 29 participants (16 pilgrims, 13 registered nurses). | Methods – Setting and Sample; Results |
| 13 | Non-participation | No participants withdrew. The number of individuals who declined was not recorded. | Methods – Data Collection |
| ***Setting*** | | | |
| 14 | Setting of data collection | Remote interviews via Zoom, within approximately one month after Hajj (20 June–11 July 2025); participants joined from their homes. | Methods – Setting and Sample; Data Collection |
| 15 | Presence of non-participants | No one other than the participant and the interviewer was present. | Methods – Data Collection |
| 16 | Description of sample | Gender, age, marital status, and nationality by group. | Results; Table 2 |
| ***Data collection*** | | | |
| 17 | Interview guide | Developed from literature and expert consultation; reviewed by expert panel; piloted with one pilgrim and one nurse; questions provided. | Methods – Data Collection; Table 1 |
| 18 | Repeat interviews | None; each participant was interviewed once. | Methods – Data Collection |
| 19 | Audio/visual recording | Audio-recorded with consent. | Methods – Data Collection |
| 20 | Field notes | Field notes taken during and after each interview. | Methods – Data Collection |
| 21 | Duration | Approximately 30–45 minutes. | Methods – Data Collection |
| 22 | Data saturation | Assessed separately for each group; reached when the final two to three interviews in each group produced no new codes. | Methods – Setting and Sample |
| 23 | Transcripts returned | Yes; participants were invited to review their transcripts (member checking). | Methods – Data Collection; Data Analysis |
| **Domain 3: Analysis and findings** | | | |
| ***Data analysis*** | | | |
| 24 | Number of data coders | Three researchers. | Methods – Data Analysis |
| 25 | Description of the coding tree | Themes and subthemes presented; development from initial codes to categories to themes described. | Methods – Data Analysis; Table 3 |
| 26 | Derivation of themes | Derived inductively from the data. | Methods – Study Design and Aims; Data Analysis |
| 27 | Software | NVivo 12.6. | Methods – Data Analysis |
| 28 | Participant checking | Participants reviewed their transcripts; findings were not returned to participants. | Methods – Data Analysis |
| ***Reporting*** | | | |
| 29 | Quotations presented | Yes; quotations are attributed to pilgrims or nurses where relevant | Results |
| 30 | Data and findings consistent | Yes; group differences and similarities summarized. | Results; Themes Summary |
| 31 | Clarity of major themes | Five major themes presented. | Results; Table 3 |
| 32 | Clarity of minor themes | Subthemes presented under each theme; divergent group perspectives in Themes Summary. | Results; Themes Summary |

Reference: Tong A, Sainsbury P, Craig J. Consolidated criteria for reporting qualitative research (COREQ): a 32-item checklist for interviews and focus groups. Int J Qual Health Care. 2007;19(6):349–357.
