# Supplementary material for: Sun protection awareness and ultraviolet protection factor product use among pilgrims and nurses during Hajj: A qualitative post-Hajj interview study
Source: PLoS One. 2026 Nov 11;21(11):e0362675. doi: 10.1371/journal.pone.0362675 (PMC13754106; doi:10.1371/journal.pone.0362675)
Supplement: Supplementary file 3 [file pone.0362675.s002.docx]

Limited Sample and Transferability

All pilgrim participants were Saudi nationals, which limits the transferability of the findings to the highly diverse international Hajj population.

Although thematic saturation was claimed, the process by which saturation was achieved for each participant group was not sufficiently described.

Lack of a Theoretical Framework

The study was not guided by any behavioral or conceptual framework (e.g., Health Belief Model, Protection Motivation Theory, or COM-B model).

Consequently, the findings remain largely descriptive and do not provide a deeper explanation of the mechanisms underlying sun-protective behaviors.

Insufficient Separation of Participant Groups

Data from pilgrims and nurses were frequently merged during analysis, making it difficult to distinguish differences and similarities between the two groups.

More comparative analysis between participants' perspectives would strengthen the study.

Limited Description of the Data Analysis Process

Although Braun and Clarke’s thematic analysis was employed, the manuscript does not clearly specify:

The number of coders involved;

Whether coding was conducted independently;

How disagreements were resolved;

How codes evolved into themes.

Incomplete Reporting According to COREQ Guidelines

Researcher reflexivity and the potential influence of the interviewer on data collection and interpretation were only briefly addressed.

Additional information regarding researchers' assumptions and strategies used to minimize bias would improve methodological transparency.

Translation Procedures Require Further Clarification

Interviews were conducted in both Arabic and English, yet the manuscript does not describe whether back-translation procedures were used to ensure semantic equivalence.

More details are needed regarding quality assurance of translated transcripts.

Inconsistencies Regarding Language and Participants

The authors mention conducting interviews with non-Arabic speakers in English, despite all pilgrims being Saudi nationals and only one nurse being non-Saudi.

This discrepancy requires clarification.

Discussion Section Is Predominantly Descriptive

The discussion largely reiterates the results instead of providing deeper interpretation and critical comparison with existing literature.

The added value and novelty of the study relative to previous research are not sufficiently emphasized.

Conclusions and Recommendations Exceed the Evidence

Recommendations such as installing vending machines for UPF products and developing specialized clothing are interesting but are not directly supported by the qualitative data.

These suggestions should be presented more cautiously.

Additional Limitations Were Not Addressed

Potential social desirability bias was not discussed.

Recall bias was not considered.

The impact of conducting interviews through Zoom on data richness and participant interaction was not addressed.

Limited Participant Characteristics

Participant demographics were restricted to age, gender, marital status, and nationality.

Information regarding education level, previous Hajj experience, professional experience of nurses, and socioeconomic status would provide a more comprehensive understanding of the sample.

Limited Use of Quotations

Some subthemes were supported by only a few quotations.

Including more diverse participant quotations would enhance the credibility and richness of the findings.

If deemed suitable, the authors may consider citing the following references, as they address related issues and may contribute to strengthening the background and discussion sections of the manuscript:

1. Perceived organisational support and university students' career exploration: the mediation role of career adaptability.

2 Involvement work systems and operational effectiveness: Exploring the moderating effect of national power distance

3 An empirical examination of individual green policy perception and green behaviors

4 How environmental knowledge management promotes employee green behavior: An empirical study

5 Culture as Context: A Five-Country Study of Discretionary Green Workplace Behavior

6 Words that matter: A cross-disciplinary investigation of importance markers in 3MT presentations

7 Measurement of China’s public health level: compilation and research of an index

8 Home-based physical activity and well-being during infectious diseases: a structural equation modeling study
