# Supplementary material for: Sun protection awareness and ultraviolet protection factor product use among pilgrims and nurses during Hajj: A qualitative post-Hajj interview study
Source: PLoS One. 2026 Nov 11;21(11):e0362675. doi: 10.1371/journal.pone.0362675 (PMC13754106; doi:10.1371/journal.pone.0362675)
Supplement: Supplementary file 4 [file pone.0362675.s005.docx]

**28-07-2026**

**Dear Editor**

Manuscript PONE-D-26-00747

Title: Sun protection awareness and ultraviolet protection factor product use among pilgrims and nurses during hajj: a qualitative interview study

We thank the Editor and the reviewers for their careful and constructive evaluation of our manuscript and their valuable feedback. We have revised the manuscript to address every point raised below.

| **Source** | **Comment** | **Response** |
| --- | --- | --- |
| **Academic Editor / Journal Requirements** | | |
| **1** | *Your ethics statement should only appear in the Methods section of your manuscript.* | Thank you for the feedback .We have moved the ethics approval and informed consent statement into a new subsection, "Ethics Approval and Consent," within the Methods section, and removed it from the Declarations section, where it previously appeared. **Line 208** |
| **2** | *The contact point for your data availability statement must not be an author on the manuscript and must be an institutional contact.* | We have revised the Data Availability and corected line 514. |
| **3** | *Please review your reference list to ensure that it is complete and correct, and that no cited works have been retracted.* | Completed. Line 532 |
| **Reviewer #1** | | |
| **1** | *It was stated that interviews were done online via ZOOM while in earlier sections it was mentioned field based contextual interviewing during hajj. It should be clarified to avoid confusion.* | Thank you for the feedback. We agree this was unclear as written. We have added a clarifying sentence at the end of the Setting and Sample section line 111 explaining that the immersive Hajj setting described refers to the context participants were reflecting on, not the mode of data collection, and revised the Data Collection section to explicitly state that interviews were conducted remotely via Zoom shortly after participants completed the 2025 Hajj season, within the stated data collection window (20/06/2025–11/07/2025), rather than during the physical rituals themselves. |
| **2** | *The author alternates between (UV protection, UPF products, UV protective products) — should be standardized.* | We have standardized terminology throughout the manuscript, using "UPF-rated products" when referring to the product category and "UV protection/exposure" when referring to the general concept. |
| **3** | *Several references need to be checked in terms of journal abbreviation and formatting style.* | We have reviewed and corrected reference formatting and journal abbreviations throughout the reference list in line with PLOS ONE style requirements. |
| **4** | *Concerns about the transparency of the methods, the depth of the analysis, and consistency in reporting (general).* | We have substantially expanded the Data Analysis section to describe the coding process in full (number of coders, independent coding procedure, disagreement resolution, and the process by which codes were grouped into the five final themes), and added a new Researcher Reflexivity subsection **line 168** describing the interviewer's background and bias-mitigation strategies. Full details are provided in the Reviewer #2 rows below, whose comments overlap substantially with this concern. |
| **Reviewer #2** | | |
| **1** | *All pilgrim participants were Saudi nationals, which limits the transferability of the findings to the highly diverse international Hajj population. Although thematic saturation was claimed, the process by which saturation was achieved for each participant group was not sufficiently described.* | We acknowledge this limitation; it is already noted in the Strengths and Limitations section line 475, which we have retained and reinforced. Regarding saturation, thematic saturation across the combined 29 interviews (16 pilgrims, 13 nurses) was assessed jointly by the research team through iterative review of emerging codes, consistent with the process described in the Setting and Sample section; no new substantive codes emerged in the final interviews of either group. |
| **2** | *The study was not guided by any behavioral or conceptual framework (e.g., Health Belief Model, Protection Motivation Theory, or COM-B model). Consequently, the findings remain largely descriptive and do not provide a deeper explanation of the mechanisms underlying sun-protective behaviors.* | We have added a new paragraph early in line 85 . |
| **3** | *Data from pilgrims and nurses were frequently merged during analysis, making it difficult to distinguish differences and similarities between the two groups. More comparative analysis between participants' perspectives would strengthen the study.* | We have added a new paragraph at the end of the Results section line 370. |
| **4** | *The manuscript does not clearly specify the number of coders involved, whether coding was conducted independently, how disagreements were resolved, and how codes evolved into themes.* | We have substantially revised the Data Analysis section line 162. |
| **5** | *Researcher reflexivity and the potential influence of the interviewer on data collection and interpretation were only briefly addressed. Additional information regarding researchers' assumptions and strategies used to minimize bias would improve methodological transparency.* | We have added a new Researcher Reflexivity subsection describing the interviewer's (MH's) clinical background line 159. |
| **6** | *Interviews were conducted in both Arabic and English, yet the manuscript does not describe whether back-translation procedures were used to ensure semantic equivalence. More details are needed regarding quality assurance of translated transcripts.* | We have revised the Data Collection section to clarify that full transcripts were translated from Arabic into English, and that a second bilingual researcher, independent of the original translation, audited a random 20% sample … line 172 |
| **7** | *The authors mention conducting interviews with non-Arabic speakers in English, despite all pilgrims being Saudi nationals and only one nurse being non-Saudi. This discrepancy requires clarification.* | We have clarified in the Data Collection section line 150 that all pilgrim participants were Saudi nationals and were interviewed in Arabic; the single non-Saudi participant (a nurse) was interviewed in English. |
| **8** | *Recommendations such as installing vending machines for UPF products and developing specialized clothing are interesting but are not directly supported by the qualitative data. These suggestions should be presented more cautiously.* | We agree and have revised the Implications section **lines 500** to reframe these suggestions explicitly as participant-generated ideas warranting feasibility piloting and evaluation, rather than as validated recommendations. |
| **9** | *Potential social desirability bias was not discussed. Recall bias was not considered. The impact of conducting interviews through Zoom on data richness and participant interaction was not addressed.* | Thanks for your valuable feedback ,We have added these three points to the Strengths and Limitations section **lines 487** |
| **10** | *Participant demographics were restricted to age, gender, marital status, and nationality. Information regarding education level, previous Hajj experience, professional experience of nurses, and socioeconomic status would provide a more comprehensive understanding of the sample.* | We agree these variables would have added useful context. As this information was not collected during data collection, we have added this as an explicit limitation in the Strengths and Limitations section **lines 488** |
| **11** | *Some subthemes were supported by only a few quotations. Including more diverse participant quotations would enhance the credibility and richness of the findings.* | Thanks for your valuable feedback We have added a "Themes Summary" section at the end of Results **lines 370**, summarizing where pilgrim and nurse perspectives converged and diverged across themes |
| **Reviewer #3** | | |
| **1** | *The authors may wish to provide additional detail regarding the coding process, specifically whether transcripts were independently coded by more than one researcher and how any differences in coding or interpretation were resolved.* | As detailed in the Reviewer #2 rows above, we have expanded the Data Analysis section to specify that three researchers independently coded transcripts against a shared codebook, with disagreements resolved through consensus discussion and, where necessary, arbitration by a senior researcher. |

We thank the Academic Editor and reviewers again for their time and constructive feedback, which we believe has substantially strengthened the manuscript.

Omar Qaladi
