# Supplementary material for: Sun protection awareness and ultraviolet protection factor product use among pilgrims and nurses during Hajj: A qualitative post-Hajj interview study
Source: PLoS One. 2026 Nov 11;21(11):e0362675. doi: 10.1371/journal.pone.0362675 (PMC13754106; doi:10.1371/journal.pone.0362675)
Supplement: Supplementary file 5 [file pone.0362675.s006.docx]

**Response to Reviewers | 4-10-2026**

**Dear Editor**

Manuscript PONE-D-26-00747

Title: *Sun protection awareness and ultraviolet protection factor product use among pilgrims and nurses during Hajj: a qualitative post-Hajj interview study*

We thank the Editor and the reviewers for their careful and constructive evaluation of our manuscript and their valuable feedback. We have revised the manuscript to address every point raised below.

| **Source** | **Comment** | **Response** |
| --- | --- | --- |
| **Academic Editor and journal requirements** | | |
| **E1** | **Remaining revision priorities**  Please revise the manuscript accordingly, with particular emphasis on resolving the   - inconsistency regarding the timing of data collection, - clarifying the distinction between heat-related illness and UV-related health effects, - and correcting the terminology related to UPF and sunscreen. - The authors should also provide clearer information on saturation across the two participant groups, - recruitment procedures, - and the evidence supporting statements such as “most participants.” | Thank you. Each point has been addressed in the manuscript (highlighted), and the details are in our responses to Reviewer #1: timing of data collection (R1.5, R1.6), heat vs UV (R1.7), UPF/sunscreen terminology (R1.8), saturation and recruitment (R1.9, with details in R1.2 and R1.3), and "most participants" (R1.10). |
| **Reviewer #1** | | |
| **R1.1** | The study included only Saudi pilgrims, despite Hajj being an international mass gathering. From my perspective the restriction might have limits transferability. kindly justify why only Saudi pilgrims were included and explain if this limitation may have influenced findings regarding language barriers, cultural beliefs, and access to sun protection. | Thank you for this important comment. We agree that including only Saudi pilgrims may limit transferability to the wider multinational Hajj population. Saudi pilgrims were intentionally selected to provide greater linguistic and cultural consistency and to establish a context-specific baseline for this exploratory qualitative study.  We have clarified this rationale in the Methods line 102- 105 and expanded the Limitations 483-490 to acknowledge that language barriers, cultural beliefs, socioeconomic factors, and access to sun-protection products may differ among international pilgrims. |
| **R1.2** | Please provide additional detail regarding participant recruitment, including how potential participants were approached, who made the initial contact, whether participation was voluntary without institutional influence. This information is particularly relevant because nurses were recruited through the Ministry of Health and participating hospitals. | Thank you for your valuble feedback. We have added a description of recruitment for each group line 106-112 and 114-120. |
| **R1.3** | The authors' claim of "representative account" should be removed. For a purposively sampled qualitative study involving 16 pilgrims and 13 nurses, "representative" is problematic. | Thank you for this comment. We agree that the term “representative” is not appropriate for a purposively sampled qualitative study. We have removed this wording and revised the sentence to emphasize the depth and diversity of perspectives rather than representativeness line 130 - 136 |
| **R1.4** | Given that this is a qualitative interview study, the authors are encouraged to assess the manuscript against the Consolidated Criteria for Reporting Qualitative Research (COREQ) 32-item checklist. Several COREQ domains, particularly researcher reflexivity, participant recruitment, saturation, data collection procedures, and analytic processes, have been addressed in the revision; however, the manuscript should be checked systematically for completeness. If appropriate, please provide the completed COREQ checklist as a supplementary file | Thank you. We checked the manuscript against all 32 COREQ items, and the completed checklist is provided as supplementary file Checklist line 663 |
| **R1.5** | Primary data are retrospective interviews, this should be integrated into the interpretation rather than merely listed as a limitation. The title/abstract should also avoid implying that the researchers directly observed behaviour during Hajj. | Thank you; we agree. The title now specifies a post-Hajj interview study line 1. The Abstract edit line 24-25 and Methods line 95-96 now state that interviews were retrospective and capture recalled, self-reported practices rather than observed behavior |
| **R1.6** | Most importantly, the manuscript contains an internal inconsistency regarding the timing of data collection, with the Methods indicating retrospective Zoom interviews after Hajj while the Strengths section states that data were collected in real time during the pilgrimage. | Thank you for identifying this error addressed in line 492-494, line 100-106 |
| **R1.7** | In addition, the manuscript should distinguish more carefully between heat-related illness and UV-related health effects | Thank you; this made the findings clearer addressed and highlited in the abstract , intro line 52-54, results line 239-241also 250-252, discussion line 427-437, conculsion line 503 – 505. |
| **R1.8** | and correct the terminology surrounding UPF and sunscreen. | Thank you. We have corrected this throughout the manscrpit and highlighted |
| **R1.9** | Further clarification is required regarding saturation across the two participant groups,  recruitment procedures, | Thank you. saturation across the two participant groups addressed in R 1.3 and also in line 141-146. Recruitment procedures addressed in R1.2. |
| **R1.10** | the evidentiary basis for statements such as “most participants.” | Thank you. Wording such as "most participants" and "many participants" has been removed or rewritten to say which group held the view (for example, "Participants in both groups were often unaware…" or "Sunscreen use was described mainly by female pilgrims"). Where participants described other pilgrims rather than themselves, Changes are highlighted throughout the manuscript." |
| **Reviewer #3** | | |
| **R3.1** | **Language and terminology**  I have reviewed the revised manuscript and the authors’ responses to the previous comments. The concerns raised in the previous round have been adequately addressed, particularly those relating to clarification of the data-collection procedures, qualitative analysis and coding, researcher reflexivity, translation procedures, comparison of participant groups, study limitations, and the framing of recommendations. The revised manuscript is clearer and methodologically better documented. There are, however, some minor English-language and terminology issues that could benefit from attention, including the broad use of “UPF” in relation to sunscreen and some grammatical awkwardness. A final general language edit would improve the presentation. I have no further substantive comments and consider the manuscript suitable for publication. | Thank you for your valuble feedback, “minor English-language” addressed  - “terminology issues that could benefit from attention, including the broad use of “UPF” in relation to sunscreen” addressed in R1.8. |
| **Reviewer #2** | | |
| **R2** | **Current review round** | No new change requested by Reviewer 2 |

We thank the Academic Editor and reviewers again for their time and constructive feedback, which we believe has substantially strengthened the manuscript.

Omar Qaladi
