# Supplementary material for: Sun protection awareness and ultraviolet protection factor product use among pilgrims and nurses during Hajj: A qualitative post-Hajj interview study
Source: PLoS One. 2026 Nov 11;21(11):e0362675. doi: 10.1371/journal.pone.0362675 (PMC13754106; doi:10.1371/journal.pone.0362675)
Supplement: Peer review history

# Peer review: Decision Letter 0

**DOI:** 10.1371/journal.pone.0362675.r001

**Authors:** Mohammed Alhoot

21 Jun 2026

Dear Dr. Qaladi,

Thank you for submitting your manuscript to PLOS ONE. After careful consideration, we feel that it has merit but does not fully meet PLOS ONE’s publication criteria as it currently stands. Therefore, we invite you to submit a revised version of the manuscript that addresses the points raised during the review process.

The manuscript covers an important and timely topic and is generally well organized. However, several issues should be addressed to improve its quality and clarity. Although two reviewers suggested only minor revisions, one reviewer raised concerns about the transparency of the methods, the depth of the analysis, and consistency in reporting. The key revisions include providing a clearer explanation of how the qualitative data were analyzed, strengthening the discussion with deeper interpretation and stronger links to relevant theories, correcting inconsistencies in terminology and descriptions of data collection, and improving the reporting of participant information and study limitations. Overall, these concerns relate mainly to the clarity and presentation of the manuscript rather than to major problems with the study design itself.

Please submit your revised manuscript by Aug 04 2026 11:59PM. If you will need more time than this to complete your revisions, please reply to this message or contact the journal office at plosone@plos.org. When you're ready to submit your revision, log on to https://www.editorialmanager.com/pone/ and select the 'Submissions Needing Revision' folder to locate your manuscript file.

- A letter that responds to each point raised by the academic editor and reviewer(s). You should upload this letter as a separate file labeled 'Response to Reviewers'.
- A marked-up copy of your manuscript that highlights changes made to the original version. You should upload this as a separate file labeled 'Revised Manuscript with Track Changes'.
- An unmarked version of your revised paper without tracked changes. You should upload this as a separate file labeled 'Manuscript'.

If you would like to make changes to your financial disclosure, please include your updated statement in your cover letter. Guidelines for resubmitting your figure files are available below the reviewer comments at the end of this letter.

If applicable, we recommend that you deposit your laboratory protocols in protocols.io to enhance the reproducibility of your results. Protocols.io assigns your protocol its own identifier (DOI) so that it can be cited independently in the future. For instructions see: https://journals.plos.org/plosone/s/submission-guidelines#loc-laboratory-protocols. Additionally, PLOS ONE offers an option for publishing peer-reviewed Lab Protocol articles, which describe protocols hosted on protocols.io. Read more information on sharing protocols at https://plos.org/protocols?utm_medium=editorial-email&utm_source=authorletters&utm_campaign=protocols.

As the corresponding author, your ORCID iD is verified in the submission system and will appear in the published article. PLOS supports the use of ORCID, and we encourage all coauthors to register for an ORCID iD and use it as well. Please encourage your coauthors to verify their ORCID iD within the submission system before final acceptance, as unverified ORCID iDs will not appear in the published article. *Only*  the individual author can complete the verification step; PLOS staff *cannot*  verify ORCID iDs on behalf of authors.

We look forward to receiving your revised manuscript.

Kind regards,

Mohammed Abdelfatah Alhoot, PhD

Academic Editor

PLOS One

Journal requirements:

When submitting your revision, we need you to address these additional requirements.

1. Please ensure that your manuscript meets PLOS ONE's style requirements, including those for file naming. The PLOS ONE style templates can be found at

https://journals.plos.org/plosone/s/file?id=wjVg/PLOSOne_formatting_sample_main_body.pdf and

https://journals.plos.org/plosone/s/file?id=ba62/PLOSOne_formatting_sample_title_authors_affiliations.pdf

2. Your ethics statement should only appear in the Methods section of your manuscript. If your ethics statement is written in any section besides the Methods, please move it to the Methods section and delete it from any other section. Please ensure that your ethics statement is included in your manuscript, as the ethics statement entered into the online submission form will not be published alongside your manuscript.

3. In the online submission form you indicate that your data is not available for proprietary reasons and have provided a contact point for accessing this data. Please note that your current contact point is a co-author on this manuscript. According to our Data Policy, the contact point must not be an author on the manuscript and must be an institutional contact, ideally not an individual. Please revise your data statement to a non-author institutional point of contact, such as a data access or ethics committee, and send this to us via return email. Please also include contact information for the third party organization, and please include the full citation of where the data can be found.

If the reviewer comments include a recommendation to cite specific previously published works, please review and evaluate these publications to determine whether they are relevant and should be cited. There is no requirement to cite these works unless the editor has indicated otherwise.

Please review your reference list to ensure that it is complete and correct. If you have cited papers that have been retracted, please include the rationale for doing so in the manuscript text, or remove these references and replace them with relevant current references. Any changes to the reference list should be mentioned in the rebuttal letter that accompanies your revised manuscript. If you need to cite a retracted article, indicate the article’s retracted status in the References list and also include a citation and full reference for the retraction notice.

[Note: HTML markup is below. Please do not edit.]

Reviewers' comments:

Reviewer's Responses to Questions

**Comments to the Author**

1. Is the manuscript technically sound, and do the data support the conclusions?

Reviewer #1: Partly

Reviewer #2: Yes

Reviewer #3: Yes

**********

2. Has the statistical analysis been performed appropriately and rigorously?

Reviewer #1: I Don't Know

Reviewer #2: Yes

Reviewer #3: Yes

**********

3. Have the authors made all data underlying the findings in their manuscript fully available?

The PLOS Data policy

Reviewer #1: No

Reviewer #2: Yes

Reviewer #3: Yes

**********

4. Is the manuscript presented in an intelligible fashion and written in standard English?

Reviewer #1: Yes

Reviewer #2: Yes

Reviewer #3: Yes

**********

Reviewer #1: The manuscript addresses an important topic which is hajj health and nursing practices during hajj, however research methodology and analysis need more clarification.

Minor grammatical errors were detected.

The author alternates between (UV protection , UPF products , UV protective products ) should be standardized.

It was stated that interviews were done online via ZOOM while in earlier sections it was mentioned field based contextual interviewing during hajj. It should be clarified to avoid confusion.

Several references need to be checked in terms of journal abbreviation and formatting style with recommendation to checked against journal guidelines.

Overall the manuscript is well organized and provides useful data for public health planning and nursing practice.

Reviewer #2: attached.

Reviewer #3: Thank you for the opportunity to review this manuscript.

This manuscript addresses an important and underexplored public health issue concerning awareness and use of sun protection measures among pilgrims and nurses during Hajj. The topic is timely and relevant given the increasing risks associated with heat exposure and ultraviolet radiation during mass gatherings.

The study employs an appropriate qualitative descriptive design. The use of purposive sampling, semi-structured interviews, thematic analysis following Braun and Clarke’s framework, and strategies to ensure trustworthiness are clearly described. Ethical approval, informed consent procedures, participant confidentiality measures, and data management procedures are appropriately reported.

The manuscript is well organized and written in clear English. The findings provide useful insights into knowledge gaps, cultural influences, barriers to sun protection, and opportunities for intervention among both pilgrims and healthcare professionals. The conclusions are supported by the findings presented and are consistent with the study objectives.

I have only a minor suggestion for clarification. The authors may wish to provide additional detail regarding the coding process, specifically whether transcripts were independently coded by more than one researcher and how any differences in coding or interpretation were resolved. This would further strengthen methodological transparency and rigor.

The Data Availability Statement appropriately addresses the confidentiality concerns associated with qualitative interview data while providing a mechanism for access by qualified researchers under appropriate ethical safeguards.

Overall, this is a well-conducted qualitative study that contributes valuable evidence to the literature on health promotion and preventive strategies during Hajj. I consider the manuscript suitable for publication following minor revision.

**********

what does this mean?). If published, this will include your full peer review and any attached files.

If you choose “no”, your identity will remain anonymous but your review may still be made public.

**Do you want your identity to be public for this peer review?**  For information about this choice, including consent withdrawal, please see our Privacy Policy

Reviewer #1: No

Reviewer #2: No

Reviewer #3: No

**********

[NOTE: If reviewer comments were submitted as an attachment file, they will be attached to this email and accessible via the submission site. Please log into your account, locate the manuscript record, and check for the action link "View Attachments". If this link does not appear, there are no attachment files.]

To ensure your figures meet our technical requirements, please review our figure guidelines: https://journals.plos.org/plosone/s/figures

You may also use PLOS’s free figure tool, NAAS, to help you prepare publication quality figures: https://journals.plos.org/plosone/s/figures#loc-tools-for-figure-preparation.

NAAS will assess whether your figures meet our technical requirements by comparing each figure against our figure specifications.

Attachment Submitted filename: 1.docx

# Author comment: Author response to Decision Letter 1

**DOI:** 10.1371/journal.pone.0362675.r002

28 Jul 2026

Thank you for the opportunity to revise manuscript We have addressed all points raised by the three reviewers and the academic editor, as detailed in our accompanying Response to Reviewers letter.

Attachment Submitted filename: Response_to_Reviewers.docx

# Peer review: Decision Letter 1

**DOI:** 10.1371/journal.pone.0362675.r003

**Authors:** Mohammed Alhoot

26 Aug 2026

Dear Dr. Qaladi,

Thank you for submitting your manuscript to PLOS One. After careful consideration, we feel that it has merit but does not fully meet PLOS One’s publication criteria as it currently stands. Therefore, we invite you to submit a revised version of the manuscript that addresses the points raised during the review process.

Please submit your revised manuscript by Oct 10 2026 11:59PM. If you will need more time than this to complete your revisions, please reply to this message or contact the journal office at plosone@plos.org. When you're ready to submit your revision, log on to https://www.editorialmanager.com/pone/ and select the 'Submissions Needing Revision' folder to locate your manuscript file.

- A letter that responds to each point raised by the academic editor and reviewer(s). You should upload this letter as a separate file labeled 'Response to Reviewers'.
- A marked-up copy of your manuscript that highlights changes made to the original version. You should upload this as a separate file labeled 'Revised Manuscript with Track Changes'.
- An unmarked version of your revised paper without tracked changes. You should upload this as a separate file labeled 'Manuscript'.

If you would like to make changes to your financial disclosure, please include your updated statement in your cover letter. Guidelines for resubmitting your figure files are available below the reviewer comments at the end of this letter.

If applicable, we recommend that you deposit your laboratory protocols in protocols.io to enhance the reproducibility of your results. Protocols.io assigns your protocol its own identifier (DOI) so that it can be cited independently in the future. For instructions see: https://journals.plos.org/plosone/s/submission-guidelines#loc-laboratory-protocols. Additionally, PLOS One offers an option for publishing peer-reviewed Lab Protocol articles, which describe protocols hosted on protocols.io. Read more information on sharing protocols at https://plos.org/protocols?utm_medium=editorial-email&utm_source=authorletters&utm_campaign=protocols.

As the corresponding author, your ORCID iD is verified in the submission system and will appear in the published article. PLOS supports the use of ORCID, and we encourage all coauthors to register for an ORCID iD and use it as well. Please encourage your coauthors to verify their ORCID iD within the submission system before final acceptance, as unverified ORCID iDs will not appear in the published article. *Only*  the individual author can complete the verification step; PLOS staff *cannot*  verify ORCID iDs on behalf of authors.

We look forward to receiving your revised manuscript.

Kind regards,

Mohammed Abdelfatah Alhoot, PhD

Academic Editor

PLOS One

Journal Requirements:

1. If the reviewer comments include a recommendation to cite specific previously published works, please review and evaluate these publications to determine whether they are relevant and should be cited. There is no requirement to cite these works unless the editor has indicated otherwise.

2. Please review your reference list to ensure that it is complete and correct. If you have cited papers that have been retracted, please include the rationale for doing so in the manuscript text, or remove these references and replace them with relevant current references. Any changes to the reference list should be mentioned in the rebuttal letter that accompanies your revised manuscript. If you need to cite a retracted article, indicate the article’s retracted status in the References list and also include a citation and full reference for the retraction notice.

Additional Editor Comments:

The authors have addressed the comments of reviewers constructively and have made several improvements to the manuscript. However, the remaining issues identified by the reviewer require careful attention before the manuscript can be considered further. Please revise the manuscript accordingly, with particular emphasis on resolving the inconsistency regarding the timing of data collection, clarifying the distinction between heat-related illness and UV-related health effects, and correcting the terminology related to UPF and sunscreen. The authors should also provide clearer information on saturation across the two participant groups, recruitment procedures, and the evidence supporting statements such as “most participants.” Please ensure that all revisions are clearly reflected in the manuscript and that the responses to the reviewer comments are complete and adequately supported.

[Note: HTML markup is below. Please do not edit.]

Reviewer's Responses to Questions

**Comments to the Author**

Reviewer #1: All comments have been addressed

Reviewer #2: (No Response)

Reviewer #3: All comments have been addressed

**********

2. Is the manuscript technically sound, and do the data support the conclusions?

Reviewer #1: Partly

Reviewer #2: (No Response)

Reviewer #3: Yes

**********

3. Has the statistical analysis been performed appropriately and rigorously?

Reviewer #1: Yes

Reviewer #2: (No Response)

Reviewer #3: N/A

**********

4. Have the authors made all data underlying the findings in their manuscript fully available?

The PLOS Data policy

Reviewer #1: No

Reviewer #2: (No Response)

Reviewer #3: No

**********

5. Is the manuscript presented in an intelligible fashion and written in standard English?

Reviewer #1: No

Reviewer #2: (No Response)

Reviewer #3: Yes

**********

Reviewer #1: The authors have responded constructively to the most previous comments. However, several issues remain that should be addressed before the manuscript can be considered for publication. Most importantly, the manuscript contains an internal inconsistency regarding the timing of data collection, with the Methods indicating retrospective Zoom interviews after Hajj while the Strengths section states that data were collected in real time during the pilgrimage. In addition, the manuscript should distinguish more carefully between heat-related illness and UV-related health effects and correct the terminology surrounding UPF and sunscreen. Further clarification is required regarding saturation across the two participant groups, recruitment procedures, and the evidentiary basis for statements such as “most participants.” Kindly address these issues.

Reviewer #2: NO

Reviewer #3: I have reviewed the revised manuscript and the authors’ responses to the previous comments. The concerns raised in the previous round have been adequately addressed, particularly those relating to clarification of the data-collection procedures, qualitative analysis and coding, researcher reflexivity, translation procedures, comparison of participant groups, study limitations, and the framing of recommendations. The revised manuscript is clearer and methodologically better documented. There are, however, some minor English-language and terminology issues that could benefit from attention, including the broad use of “UPF” in relation to sunscreen and some grammatical awkwardness. A final general language edit would improve the presentation. I have no further substantive comments and consider the manuscript suitable for publication.

**********

what does this mean?). If published, this will include your full peer review and any attached files.

If you choose “no”, your identity will remain anonymous but your review may still be made public.

**Do you want your identity to be public for this peer review?**  For information about this choice, including consent withdrawal, please see our Privacy Policy

Reviewer #1: No

Reviewer #2: No

Reviewer #3: No

**********

[NOTE: If reviewer comments were submitted as an attachment file, they will be attached to this email and accessible via the submission site. Please log into your account, locate the manuscript record, and check for the action link "View Attachments". If this link does not appear, there are no attachment files.]

To ensure your figures meet our technical requirements, please review our figure guidelines: https://journals.plos.org/plosone/s/figures

You may also use PLOS’s free figure tool, NAAS, to help you prepare publication quality figures: https://journals.plos.org/plosone/s/figures#loc-tools-for-figure-preparation.

NAAS will assess whether your figures meet our technical requirements by comparing each figure against our figure specifications.

Attachment Submitted filename: Dr Nihal.pdf

# Author comment: Author response to Decision Letter 2

**DOI:** 10.1371/journal.pone.0362675.r004

5 Oct 2026

Thank you for the opportunity to revise manuscript We have addressed all points raised by the three reviewers and the academic editor, as detailed in Response to Reviewers file.

Attachment Submitted filename: Response_to_Reviewers_Columns_04_10_2026.docx

# Peer review: Decision Letter 2

**DOI:** 10.1371/journal.pone.0362675.r005

**Authors:** Mohammed Alhoot

29 Oct 2026

Sun protection awareness and ultraviolet protection factor product use among pilgrims and nurses during Hajj: a qualitative post-Hajj interview study

PONE-D-26-00747R2

Dear Dr. Qaladi,

We’re pleased to inform you that your manuscript has been judged scientifically suitable for publication and will be formally accepted for publication once it meets all outstanding technical requirements.

Within one week, you’ll receive an e-mail detailing the required amendments. When these have been addressed, you’ll receive a formal acceptance letter and your manuscript will be scheduled for publication.

An invoice will be generated when your article is formally accepted. Please note, if your institution has a publishing partnership with PLOS and your article meets the relevant criteria, all or part of your publication costs will be covered. Please make sure your user information is up-to-date by logging into Editorial Manager at Editorial Manager® and clicking the ‘Update My Information' link at the top of the page. For questions related to billing, please contact billing support.

If your institution or institutions have a press office, please notify them about your upcoming paper to help maximize its impact. If they’ll be preparing press materials, please inform our press team as soon as possible -- no later than 48 hours after receiving the formal acceptance. Your manuscript will remain under strict press embargo until 2 pm Eastern Time on the date of publication. For more information, please contact onepress@plos.org.

Kind regards,

Mohammed Abdelfatah Alhoot, PhD

Academic Editor

PLOS One

Additional Editor Comments (optional):

Reviewers' comments:

Reviewer's Responses to Questions

**Comments to the Author**

Reviewer #1: All comments have been addressed

**********

2. Is the manuscript technically sound, and do the data support the conclusions?

Reviewer #1: Partly

**********

3. Has the statistical analysis been performed appropriately and rigorously?

Reviewer #1: Yes

**********

4. Have the authors made all data underlying the findings in their manuscript fully available?

The PLOS Data policy

Reviewer #1: Yes

**********

5. Is the manuscript presented in an intelligible fashion and written in standard English?

Reviewer #1: Yes

**********

Reviewer #1: The authors have satisfactorily addressed the comments and made the necessary revisions to improve the quality and clarity of the manuscript. I have no further comments or recommendations and support its acceptance for publication in the journal.

**********

what does this mean?). If published, this will include your full peer review and any attached files.

If you choose “no”, your identity will remain anonymous but your review may still be made public.

**Do you want your identity to be public for this peer review?**  For information about this choice, including consent withdrawal, please see our Privacy Policy

Reviewer #1: **Yes:**  Dr Nihal Ahmed Hanafy

**********

# Editor report: Acceptance letter

**DOI:** 10.1371/journal.pone.0362675.r006

**Authors:** Mohammed Alhoot

PONE-D-26-00747R2

PLOS One

Dear Dr. Qaladi,

I'm pleased to inform you that your manuscript has been deemed suitable for publication in PLOS One. Congratulations! Your manuscript is now being handed over to our production team.

At this stage, our production department will prepare your paper for publication. This includes ensuring the following:

* All references, tables, and figures are properly cited

* All relevant supporting information is included in the manuscript submission,

* There are no issues that prevent the paper from being properly typeset

You will receive further instructions from the production team, including instructions on how to review your proof when it is ready. Please keep in mind that we are working through a large volume of accepted articles, so please give us a few days to review your paper and let you know the next and final steps.

Lastly, if your institution or institutions have a press office, please let them know about your upcoming paper now to help maximize its impact. If they'll be preparing press materials, please inform our press team within the next 48 hours. Your manuscript will remain under strict press embargo until 2 pm Eastern Time on the date of publication. For more information, please contact onepress@plos.org.

You will receive an invoice from PLOS for your publication fee after your manuscript has reached the completed accept phase. If you receive an email requesting payment before acceptance or for any other service, this may be a phishing scheme. Learn how to identify phishing emails and protect your accounts at https://explore.plos.org/phishing.

If we can help with anything else, please email us at customercare@plos.org.

Thank you for submitting your work to PLOS One and supporting open access.

Kind regards,

PLOS One Editorial Office Staff

on behalf of

AP. Dr. Mohammed Abdelfatah Alhoot

Academic Editor

PLOS One
